# Impact of Soil Microbes and Oxygen Availability on Bacterial Community Structure of Decomposing Poultry Carcasses

**DOI:** 10.3390/ani11102937

**Published:** 2021-10-11

**Authors:** Michelle A. Miguel, Seon-Ho Kim, Sang-Suk Lee, Yong-Il Cho

**Affiliations:** Department of Animal Science and Technology, Sunchon National University, Suncheon 57922, Korea; mamiguel@scnu.ac.kr (M.A.M.); mhs0425@scnu.ac.kr (S.-H.K.); rumen@scnu.ac.kr (S.-S.L.)

**Keywords:** 16S rRNA, ASV, bacterial diversity, carcass, decomposition, MiSeq, poultry

## Abstract

**Simple Summary:**

Decomposition is a complex process that involves several factors, such as temperature, pH, humidity, and microbes. Microbes play a significant role in the carcass decomposition process. Microcosm burial set-ups were prepared and poultry carcasses were decomposed for 60 days in unsterilized and sterilized soil, incubated under aerobic or anaerobic conditions. The moisture content, pH, alpha and beta diversity were affected by the soil microbial community and oxygen availability during the decomposition of poultry carcasses. The bacterial taxa composition was also altered during the poultry carcass decomposition. These changes suggested that the soil with an intact microbial community and oxygen availability influenced the bacterial community structure during the decomposition of poultry carcasses. The results of this study provided information on the different bacterial species which might be associated with the decomposition of poultry carcasses.

**Abstract:**

The impact of soil with an intact microbial community and oxygen availability on moisture content, soil pH, and bacterial communities during decomposition of poultry carcasses was investigated. Poultry carcasses were decomposed in soil with or without a microbial community, under aerobic or anaerobic conditions. The samples collected in each microcosm burial set-up were analyzed by targeted 16S rRNA amplicon sequencing and Amplicon sequence variants (ASV) method. Our results showed that moisture was high in the burial set-ups under anaerobic conditions and pH was high in the burial set-ups under aerobic conditions. Meanwhile, the Chao1 and Shannon index significantly differed between the different burial set-ups and across different time points. In addition, bacterial taxa composition during the early period of decomposition differed from that of the late period. A total of 23 phyla, 901 genera, and 1992 species were identified. Firmicutes was the most dominant phyla in all burial set-ups throughout the decomposition. At day 60, *Pseudogracilibacillus* was dominant in the burial set-ups under aerobic conditions, while *Lentibacillus* dominated in the burial set-ups under anaerobic conditions. This study demonstrated that the soil microbial community and availability of oxygen significantly affected the changes in moisture content, pH, and bacterial composition during the decomposition process.

## 1. Introduction

The outbreak of highly contagious diseases, such as avian influenza has been of great concern in many countries [1,2,3,4,5]. The disposal of the infected and potentially infected animals is necessary, specifically for disease containment [6]. Disposal options for such animals include burial, incineration, composting, rendering, lactic acid fermentation, alkaline hydrolysis, and anaerobic digestion [7,8]. Among these disposal methods, burial, composting, rendering and incineration are the commonly utilized methods [9]. Decomposition causes significant and sequential changes in the bacterial communities within the soil, and which is often correlated with changes in the stage of decomposition. The decomposition process is affected by several environmental factors, such as temperature, humidity, pH and the cadaver itself [10,11,12,13,14]. Decomposition can also be affected by soil microorganisms and oxygen availability. Decomposition of buried carcasses mainly relies on the activity of microorganisms producing extracellular proteolytic enzymes which break the polymers of organic matter into oligomeric and monomeric molecules [15]. The decomposing carcass contributes nutrients to the soil through nutrient leaching, which affects the microbial communities in the soil near the carcass and surrounding environment [10,16]. Microbial activity in, on, and around carcasses is recognized as a crucial factor that can affect the decomposition rate [17,18,19]. 

Soil microorganisms play an important role in the decomposition process as they synthesize proteases in response to an animal organic matter supply and can degrade prion proteins in vitro [20,21]. Microbial communities are important in maintaining soil quality due to their involvement in organic matter dynamics, nutrient cycling, pathogenic spread and decomposition [16,17,22,23,24]. Several studies have demonstrated, metabolites from decomposing carcasses can affect the land and surrounding environment [16,25]. Heat is known to inactivate enzymes secreted by soil microorganisms. The removal of soil microorganisms by autoclaving typically results in a decrease in microbial biomass and enzyme activity [26,27]. Meanwhile, the access or restriction of oxygen content on the body is an important factor in the decomposition rate [28].

Studies have been conducted to investigate whether the presence of an endogenous soil microbial community will influence carcass decomposition. However, the composition of microbial communities between soil with, or without the microbial communities, and decomposed aerobically, or anaerobically has not been clearly elucidated. Therefore, an investigation on changes to bacterial communities in animal burial soil is necessary to identify the possible pathogenic microorganisms which can cause contamination of the environment and a risk to public health. Thus, the present study investigated the changes in the moisture content, pH, and bacterial communities of decomposing poultry carcasses buried in either (i) soil with an intact microbial community (unsterilized soil) or (ii) soil that was sterilized and incubated under aerobic or anaerobic conditions for 60 days.

## 2. Materials and Methods

### 2.1. Ethical Statement 

All experimental protocol of this study was approved by the Animal Care and Use Committee (Approval number: SCNU IACUC-2019-7) of the Sunchon National University (Suncheon, Jeollanam-do, Korea). All experiments were performed as per the guidelines and regulations set by this governing body. 

### 2.2. Carcass and Soil Preparation

Two Hy-Line Brown hens, weighing 2.50 ± 0.1 kg, were purchased commercially and used in the study. The Hy-Line Brown hens were euthanized and handled as per the guidelines of the Institutional Animal Care and Use Committee of the Sunchon National University. The carcasses were separated from the bones, and the chicken remains, including feathers, internal organs, muscular, epithelial, and visceral tissues, were crushed and homogenized. 

Approximately 10 kg of soil was obtained from an agricultural field in the experimental farm of the Sunchon National University. The soil was sieved through a 2 mm sieve to remove crop roots and stones and divided into two parts. One part of the collected soil was sterilized (S), while the other part was kept unsterilized (soil with an intact microbial community, U). To prepare the sterilized soil, the sample was placed in a polypropylene bag and autoclaved at 121 °C, 15 psi thrice in four days to destroy microbes, fungi and their spores [11].

### 2.3. In Vitro Set-Up for Carcass Decomposition 

**Figure 1:**
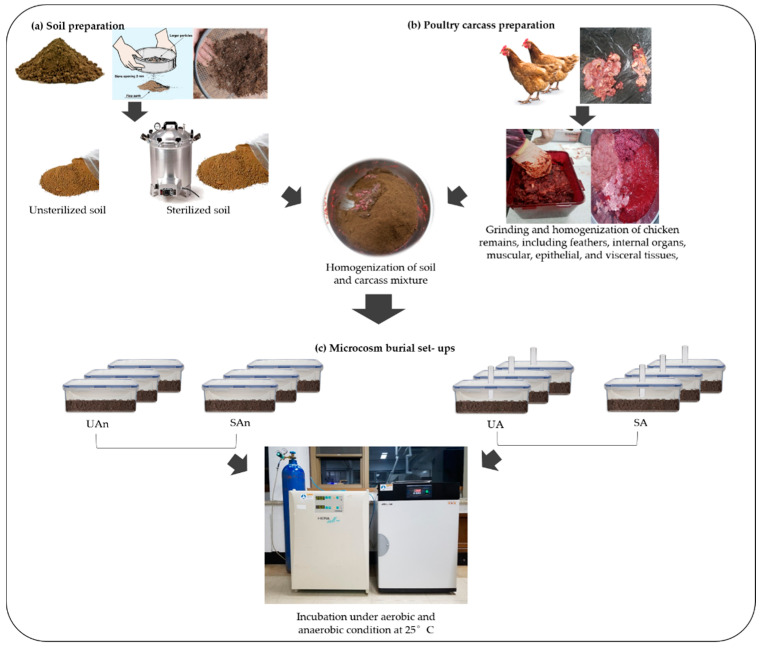
Schematic diagram of the experimental setup. UA, unsterilized soil–aerobic condition; SA, sterilized soil–aerobic condition; UAn, unsterilized soil–anaerobic condition and SAn, sterilized soil–anaerobic condition.

A 2 × 2 microcosm burial set-up was made using two types of soil (i. soil with an intact microbial community; ii. sterilized soil) under two incubation conditions (a. with oxygen access; b. without oxygen access) (Figure 1). The four microcosm burial set-ups were as follows: UA (unsterilized soil–aerobic condition), SA (sterilized soil–aerobic condition), UAn (unsterilized soil–anaerobic condition), and SAn (sterilized soil–anaerobic condition). Three replicates for each burial set-up incubated at different periods (0, 5, 10, 30, and 60 days) were prepared. A total of sixty sterilized containers (450 mL each) were used, with each containing 112.5 g of soil and 37.5 g of meat. The mixture of soil and meat was decomposed under aerobic and anaerobic conditions. Anaerobic conditions were created by sealing all parts of the container and placing it in an incubator with 5% flowing CO_2_ gas. For simulating aerobic conditions, the lid of the reactor was punctured so that air could pass through the hole before placing it in an incubator. All experimental set-ups were incubated at 25 °C for a total period of 60 days.

### 2.4. Sample Collection

Samples were collected on days: 0, 5, 10, 30 and 60. Day 0 was the initial placement day followed by subsequent sample collections on days 5, 10, 30 and 60. Individual samples (approx. 30 g) were taken from each replicate container for each period and distributed into two sterile 50 mL Falcon^®^ tubes. One tube was stored at 4 °C until physicochemical analysis, and the other tube was stored at −80 °C for further molecular analysis.

### 2.5. Moisture Content and pH during Carcass Decomposition

The pH was measured by suspending 1 g of soil in 5 mL of sterilized distilled water followed by vortexing for 1 min. The large particles from the mixture were allowed to settle for 5 min and the supernatant was collected and its pH was measured with a pH meter (SevenCompact™ pH/Ion meter S220, Mettler Toledo, Switzerland). The average readings of the three samples were used to estimate the pH for each soil sample [11]. The moisture content of the soil and carcass mixture was estimated according to the standard method AS 1289 B1.1. One gram of burial soil was weighed, placed in an aluminum plate, and oven-dried overnight at 105 °C.

### 2.6. DNA Extraction, PCR Amplification and 16S rRNA Amplicon Sequencing

The DNA was extracted from 0.25 g of soil samples obtained from each burial set-up using the DNeasy^®^ PowerSoil^®^ Kit (Qiagen, Hilden, Germany) according to the manufacturer’s instructions. The extracted DNA was stored at −20 °C until further processing. The quality and quantity of the extracted DNA were checked using Quant-IT PicoGreen (Invitrogen, Grand Island, NY, USA). DNA sequencing libraries targeting the V3–V4 hypervariable region of the 16S rRNA gene were performed according to the Illumina 16S metagenomic sequencing library preparation method [29]. This consists of two PCR steps. In the first amplification, specific primers were used, while in the second, index information for sample identification was added. The DNA was amplified by primary PCR using universal primer pair with Illumina adapter overhang sequences, S-D-Bact-0341-b-S-1 (5′–TCG TCG GCA GCG TCA GAT GTG TAT AAG AGA CAG CCT ACG GGN GGC WGC A–3′) and S-D-Bact-0785-a-A-21 (5′–GTC TCG TGG GCT CGG AGA TGT GTA TAA GAG ACA GGA CTA CHV GGG TAT CTA ATC C–3′) [15]. The PCR was performed with 2.5 μL of DNA sample (5 ng/μL), 5 μL each of the universal forward and reverse primer, and 12.5 μL of 2× KAPA HiFi HotStart ReadyMix (KAPA Biosystems, Wilmington, MA, USA) in a total volume of 25 μL. The cycle condition comprised an initial denaturation at 95 °C for 3 min, followed by 25 cycles of denaturation at 95 °C for 30 s, annealing at 55 °C for 30 s, and extension at 72 °C for 30 s, and a final extension at 72 °C for 5 min. The PCR products were purified with AMPure XP beads (Agencourt Bioscience, Beverly, MA, USA) to remove free primers and primer-dimer species. Following purification, 2 uL of the primary PCR product was amplified for secondary PCR for library construction. Primer sequences used for the secondary PCR are as follows: a Nextera XT Index primer pair (Illumina^®^, USA), Primer 1 (N7xx): 5′–AAT GAT ACG GCG ACC ACC GAG ATC TAC AC–[i5]–TCG TCG GCA GCG TC–3′ and Primer 2 (S5xx): 5′–CAA GCA GAA GAC GGC ATA CGA GAT–[i7]–GTC TCG TGG GCT CGG–3′. The PCR consisted of 5 μL of sample DNA, 5 μL each of Nextera XT Index primers 1 and 2, 25 μL of 2× KAPA HiFi HotStart ReadyMix (KAPA Biosystems, Wilmington, MA, USA), and 10 μL of PCR Grade Water. The cycle conditions comprised an initial denaturation at 95 °C for 3 min, followed by 8 cycles of denaturation at 95 °C for 30 s, annealing at 55 °C for 30 s, and extension at 72 °C for 30 s, and a final extension at 72 °C for 5 min. The final PCR product (final library) was cleaned up before quantification using AMPure XP beads (Agencourt Bioscience, Beverly, MA). Finally, the PCR products were quantified using qPCR according to the qPCR Quantification Protocol Guide (KAPA Library Quantification Kit for Illumina Sequencing platforms) and library quality was assessed using the TapeStation D1000 ScreenTape (Agilent Technologies, Waldbronn, Germany). Equimolar amounts of the barcoded V3–V4 amplicons were pooled and paired-end sequenced (2 × 300 bp) on an Illumina MiSeq^®^ platform (Illumina Inc., San Diego, CA, USA) using v3 reagents, according to the manufacturer’s instructions at the Macrogen Inc. (Seoul, Korea).

### 2.7. Sequence Data Processing and Metataxonomic Analysis

After sequencing was completed, Illumina MiSeq raw data was classified by sample using an index sequence, and a paired-end FASTQ file was generated for each sample. Sequences were demultiplexed, and barcodes and adaptors were removed using the Cutadapt v3.2 program [30].

Denoising strategies were applied to obtain amplicon sequence variants (ASVs) using the Divisive Amplicon Denoising Algorithm 2 (DADA2) v1.18.0 [31] in the R program v4.0.3. For paired-end read, forward reads were truncated at 250 bp, reverse reads were truncated at 200 bp, and sequences with expected errors of 2 or more were excluded. Then, the error model for each batch was established to remove the noise for each sample. After assembling the paired-end sequence corrected for sequencing error into one sequence, the Chimera sequence was removed using the DADA2 Consensus method to form ASVs. In addition, for comparative analysis of the microbial community, the QIIME v1.9 [32] program was used to normalize by applying subsampling based on the number of reads of the sample with the minimum number of reads among all samples.

Taxonomy was assigned to ASVs using the BLAST+ v2.9.0 [33] against the Reference Database (NCBI 16S Microbial DB). The taxonomy information for the organism of the subject with the highest similarity was assigned. At this time, if the query coverage of the best hit matching the database is less than 85% or the identity of the matched area is less than 85%, taxonomy information is not allocated.

Using QIIME with the above ASVs abundance and taxonomy information, a comparative analysis of various microbial communities was performed. The ASV abundance was normalized by rarefying each sample such that all the samples had the same number of total counts (25,610 reads). Alpha diversity was assessed by Chao1 and Shannon index. Based on the weighted and unweighted UniFrac distances, beta diversity between samples (information on microbial community diversity among samples in the comparison group) was obtained, and the relationship between samples was visualized through PCoA.

### 2.8. Statistical Analysis

Data were analyzed using the general linear model (GLM) procedure of Statistical Analysis Systems (SAS) version 9.4 (SAS Institute Inc., Cary, NC, USA). All variables were tested for normality using the Shapiro–Wilk test. We used analysis of variance (ANOVA) to test for effects of different burial set-ups (UA, SA, UAn, and SAn), decomposition day (0, 5, 10, 30, and 60), and their interaction. The model used was:

Y_ijk_ = µ + α_i_ + β_j_ + (αβ)_ij_ + ϵ_ijk_

where, Y_ijk_ is the response variable; µ is the overall mean; α_i_ is the main effect of different burial set-ups (B); β_j_ is the main effect of decomposition day (D); (αβ)_ij_ is the interaction between the different burial set-up and decomposition day (B × D) and ϵ_ijk_ is the random error of the k^th^ observation from the (*i*, *j*, *k*)^th^ treatment.

Differences of moisture, pH, and alpha diversity index between burial set-ups on day 0, 5, 10, 30, and 60 were assessed using Tukey’s honestly significant difference (HSD) post hoc test. A *p*-value < 0.05 was considered statistically significant. Beta diversity was calculated based on the unweighted and weighted UniFrac distance matrix and the ordination plot was visualized using principal coordinate analysis (PCoA). We used permutational analysis of variance (PERMANOVA) to determine significant differences in beta diversity. A Venn diagram of the membership-based representation of unique, shared, and core bacterial community were generated using jvenn [34]. The bacterial abundance profiles were calculated at phylum, genus, and species levels and were plotted as a bar graph. Linear discriminant analysis Effect Size (LEfSe) was performed to determine the bacterial taxa that most likely explained differences between burial set-ups. For LEfSe analysis, only taxa (species level) with an LDA score of >2.0 and a *p*-value of 0.05, as determined by the Kruskal–Wallis rank-sum test, are shown.

## 3. Results

### 3.1. Effect of Oxygen Availability and Sterilized Soil in Moisture Content and pH during Carcass Decomposition

**Figure 2:**
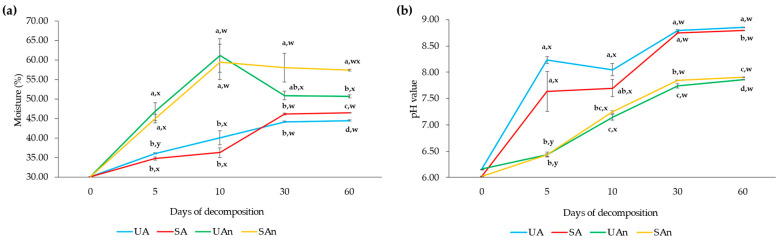
Changes in the (**a**) moisture content (%) and (**b**) pH during decomposition of poultry carcasses. UA, SA, UAn, and SAn represent unsterilized soil–aerobic condition, sterilized soil–aerobic condition, unsterilized soil–anaerobic condition, and sterilized soil–anaerobic condition, respectively. The 0, 5, 10, 30, and 60 represent the sampling period. Different letters in the figure (a–d) indicate the significant differences (*p* < 0.05) between burial set-ups at each sampling day, while (w–z) indicate the significant differences (*p* < 0.05) between the sampling periods of each burial set-up.

The moisture content and pH demonstrated significant differences (*p* < 0.05) between burial set-ups, days of decomposition, as well as the interaction of burial set-ups and decomposition day (Figure 2; Table S1). Moisture content ranged between 36.05% to 61.16% and became relatively constant from day 30 (Figure 2a). The moisture significantly varied between burial groups (*p* < 0.05) and across different time points (*p* < 0.05). Burial set-ups under anaerobic conditions (UAn and SAn) had a higher moisture content compared to those in aerobic conditions. The burial set-ups (UAn and SAn) in anaerobic conditions have high moisture at days 5 to 10, decreased from day 30 and became relatively stable until day 60. Meanwhile, the moisture in the burial set-ups (UA and SA) under aerobic conditions tended to increase until day 30 and become stable until day 60. On day 60 of decomposition, higher moisture was observed in the sterilized soil-anaerobic burial set-up.

The pH significantly differs between burial groups (*p* < 0.05) and across different sampling days (*p* < 0.05) (Figure 2b; Table S1). The pH of burial set-ups UA and SA under aerobic conditions was higher (*p* < 0.05) than those in anaerobic conditions. The pH in UA and SA decreased on day 10 and then increased on day 30 and become relatively stable until day 60. Meanwhile, the pH in UAn and SAn increased until day 30 and then become stable until day 60. In addition, the pH of the samples increased as the decomposition process progressed and remained relatively constant from days 30 to 60. At the end of day 60 of decomposition, a higher pH (*p* < 0.05) was observed in both the sterilized and unsterilized soil-aerobic condition burial set-ups (SA and UA) compared to other burial set-ups.

### 3.2. Species Richness and Diversity of Bacterial Community in Different Burial Set-Ups

**Figure 3:**
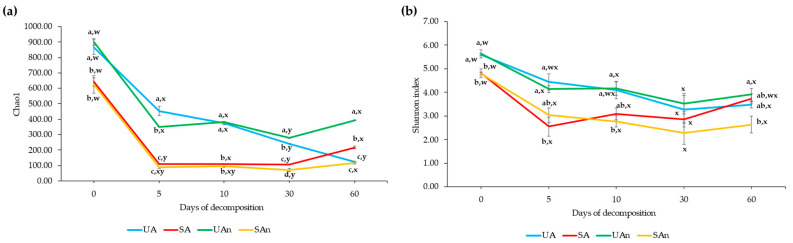
Alpha diversity indices of decomposing poultry carcasses in different burial set-ups decomposed for 60 days. (**a**) Chao1 and (**b**) Shannon index. UA, SA, UAn, and SAn represent unsterilized soil–aerobic condition, sterilized soil–aerobic condition, unsterilized soil–anaerobic condition, and sterilized soil–anaerobic condition, respectively. The 0, 5, 10, 30, and 60 represent the sampling period. ANOVA was used to test for the overall comparison of the Chao1 and Shannon among the burial set-ups on days 0, 5, 10, 30, and 60 of decomposition. Different letters in the figure (a–d) indicate the significant differences (*p* < 0.05) between burial set-ups at each sampling day, while (w–z) indicate the significant differences (*p* < 0.05) between the sampling period of each burial set-up.

A total of 6578 ASVs were obtained from all samples through the Illumina sequencing analysis. Rarefaction curves revealed that all samples were sequenced to sufficient depth to achieve asymptote, indicating a good representation of the microbial community (Figure S1). Burial set-ups, days of decomposition, and their interaction affect significantly both Chao1 and Shannon indexes (Figure 3; Table S2).

Chao1 differed significantly between burial set-ups at all time points (*p* < 0.05) (Figure 3a). During the initial day (day 0), Chao1 was significantly higher in UA and UAn burial set-ups compared to SA and SAn burial set-ups (*p* < 0.05). On day 5, it was significantly higher in UA compared to SA, UAn, and SAn burial set-ups (*p* < 0.05). On day 10, Chao1 was significantly higher in UA and UAn burial set-ups compared to SA and SAn burial set-ups (*p* < 0.05). Meanwhile, Chao1 varied significantly between the burial set-ups on day 30 (*p* < 0.05), with UAn burial set-up having the highest Chao1. On the other hand, Chao1 was significantly higher in UAn compared to other burial set-ups on day 60 (*p* < 0.05). We observed that the Chao1 in UA burial set-up significantly decreased as decomposition progressed, while UAn, SA, and SAn tended to decrease from days 5 to 30 and increased in day 60 (*p* < 0.05).

The Shannon index differed significantly between burial set-up, day of decomposition, and their interaction (*p* < 0.05) (Table S2). The Shannon index significantly varied between burial set-ups at days 0, 5, 10, and 60 (*p*-value = 0.0022, 0.0092, 0.0209, and 0.0297, respectively) (Figure 3b). Burial set-ups UA and UAn had a significantly higher Shannon index compared to SA and SAn burial set-ups at days 0, 5, and 10 (*p* < 0.05). Meanwhile, no significant difference was found among the burial set-ups on day 30, however, the Shannon index decreased on this day. On day 60, the Shannon index increased, with UAn burial set-up having the highest Shannon index among the other burial set-ups (*p* = 0.0297).

**Figure 4:**
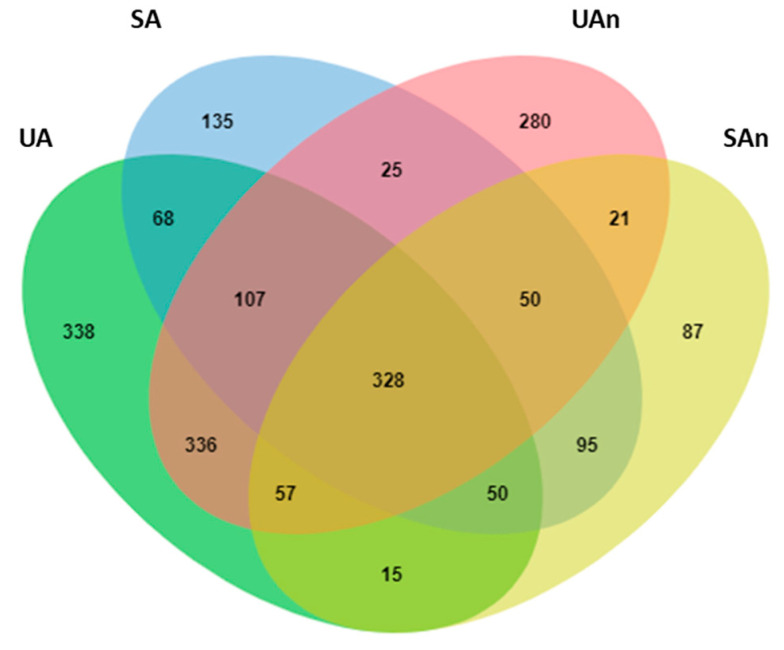
Venn diagram of shared and unique bacterial species in the different burial set-ups during poultry carcass decomposition. UA, unsterilized soil–aerobic condition; SA, sterilized soil–aerobic condition; UAn, unsterilized soil–anaerobic condition and SAn, sterilized soil–anaerobic condition.

The Venn diagram showed the similarities and differences between the communities in the different burial set-ups (Figure 4; Tables S3 and S4). The shared taxa by all samples in each burial set-up were deemed to be core bacterial communities. From 1992 ASVs, 328 bacterial ASVs were shared among all burial set-ups. Meanwhile, a total of 824 ASVs were identical in two or three groups, while 338, 135, 280, and 87 ASVs were unique to UA, SA, UAn, and SAn burial set-ups, respectively.

**Figure 5:**
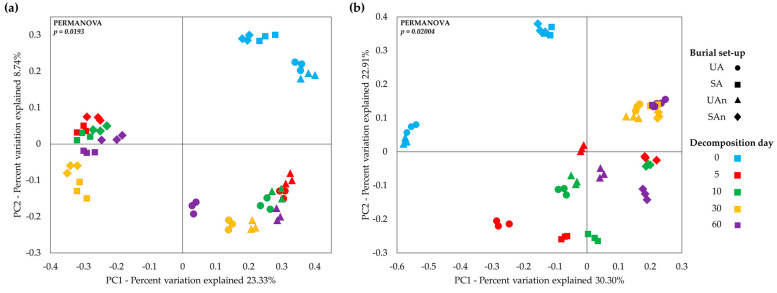
Two-dimensional principal coordinate analysis (PCoA) plot of (**a**) Unweighted and (**b**) Weighted UniFrac distance matrix of bacterial community during carcass decomposition.

Based on the unweighted PCoA, the principal component 1 (PC1) and PC2 analysis showed 23.33% and 8.74% of variance explained, respectively (Figure 5a). The bacterial communities were clustered based on the unsterilized and sterilized burial group set-up and by decomposition period. The unsterilized soil group (UA and UAn) at the initial day of decomposition formed a distinct cluster, separate from the other samples. Similarly, the samples in the sterilized soil group (SA and SAn) formed a cluster together and separated from the other samples. This indicates that the bacterial composition from these clusters was different from those in days 5 to 60. Meanwhile, the bacterial community at days 5, 10, 30, and 60 of UA and UAn were clustered together. Likewise, SA and SAn burial groups were clustered together at days 5, 10, 30, and 60. On the other hand, weighted PCoA revealed 30.30% and 22.91% variation for PC1 and PC2, respectively (Figure 5b). The plot showed that the samples from SA and SAn formed a distinct cluster. Similarly, UA and UAn also formed a cluster together. Meanwhile, the bacterial communities of different burial set-ups were similar at day 30. This indicates that the bacterial composition from those clusters was likely composed of similar microorganisms.

### 3.3. Bacterial Community Composition during Poultry Carcass Decomposition

A total of 23 phyla, 61 classes, 133 orders, 293 families, 901 genera, and 1992 species were identified in the samples. The bacterial community was mainly comprised of Firmicutes, Proteobacteria, and Actinobacteria, with a relative abundance of 77.53%, 11.53% and 6.76% of the total ASVs in all samples, respectively.

**Figure 6:**
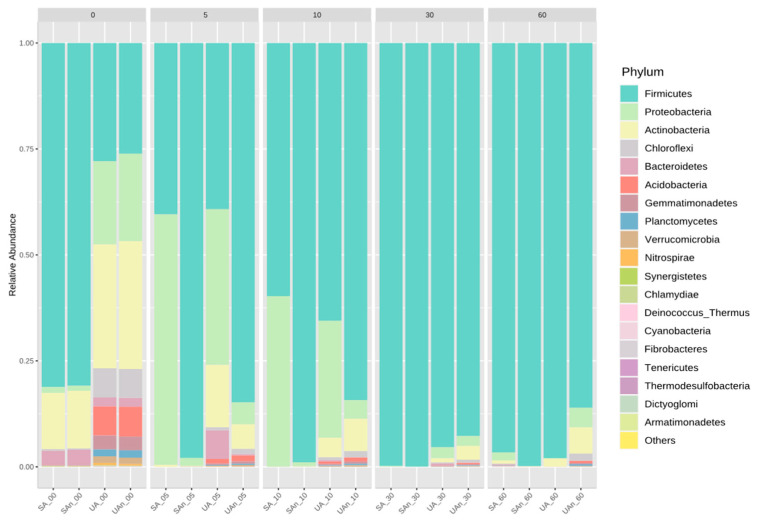
Relative abundance at phylum level during decomposition of poultry carcass. “Others” represent taxa with counts <10. UA, unsterilized soil–aerobic condition; SA, sterilized soil–aerobic condition; UAn, unsterilized soil–anaerobic condition and SAn, sterilized soil–anaerobic condition. The 0, 5, 10, 30, and 60 represent the sampling period.

On the initial day (day 0), Firmicutes dominated the SA and SAn burial set-ups, with the relative abundance of 81.07% and 80.79%, respectively (Figure 6). Meanwhile, UA and UAn were mostly composed of Firmicutes, Proteobacteria, Actinobacteria, Acidobacteria, Chloroflexi, and Bacteroidetes. At the early stage of decomposition (day 5), the abundance of Firmicutes increased in the UAn, and SAn burial set-ups but decreased in SA and UA burial set-ups. In contrast, Proteobacteria increased in UA, but decreased in UAn, SA, and SAn. In the following days (10–60), an increase of Firmicutes and a decrease of Proteobacteria could be observed. Meanwhile, Actinobacteria is more abundant in burial set-ups with unsterilized soil than with sterilized soil. In addition, the abundance of Actinobacteria tended to decrease from day 5 in all burial set-ups.

**Figure 7:**
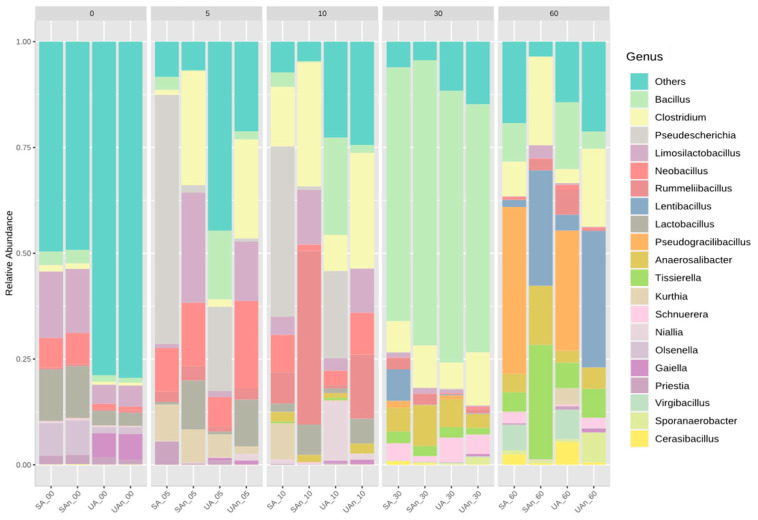
Relative abundance of the top 20 ASVs at genus level during decomposition of poultry carcass. “Others” represent taxa with counts < 10. UA, unsterilized soil–aerobic condition; SA, sterilized soil–aerobic condition; UAn, unsterilized soil–anaerobic condition and SAn, sterilized soil–anaerobic condition. The 0, 5, 10, 30, and 60 represent the sampling period.

At the genus level, Bacillus, Clostridium, Pseudescherichia, Limosilactobacillus, Neobacillus, Rummeliibacillus, Lentibacillus, Lactobacillus, Pseudogracilibacillus, and Anaerosalibacter were the most common genera in the decomposing carcass (Figure 7). On the initial day, Limosilactobacillus and Lactobacillus were the most abundant genera in all burial set-ups. Meanwhile, Gaiella was abundant in burial set-ups with unsterilized soil (UA and UAn) on the initial day. However, the relative abundance of Gaiella in those set-ups decreased as the decomposition progressed.

In the UA burial set-up, *Pseudescherichia* and *Neobacillus* predominated the bacterial community on days 5 and 10; *Bacillus* (64.18%) on day 30 and *Pseudogracilibacillus* (27.81%) and *Bacillus* (15.54%) on day 60 of decomposition. In the UAn burial set-up, *Clostridium* (23.36%), *Neobacillus* (20.58%), *Limosilactobacillus* (14.08%), and *Lactobacillus* (11.02%) were dominant on day 5; *Clostridium* (27.23%), *Rummeliibacillus* (15.07%), and *Limosilactobacillus* (10.41%) on day 10; *Bacillus* (58.27%) and *Clostridium* (12.51%) on day 30 and *Lentibacillus* and *Clostridium* (18.32%) on day 60 of decomposition. In the SA burial set-up, *Pseudescherichia* (58.62%) and *Neobacillus* (10.22%) was dominant on day 5; *Pseudescherichia* (40.07%) and *Clostridium* (14.02%) on day 10; *Bacillus* (59.91%) on day 30 and *Pseudogracilibacillus* (39.08%) on day 60 of decomposition. In the SAn burial set-up, *Clostridium* (26.93%), *Limosilactobacillus* (26.04%), and *Neobacillus* (15.01%), and *Lactobacillus* (11.52%) were dominant on day 5; *Rummeliibacillus* (40.94%)*, Clostridium* (29.27%), and *Limosilactobacillus* (12.94%) on day 10; *Bacillus* (67.34%) and *Clostridium* (10.05 %) on day 30 and *Lentibacillus* (27.27%), Tissierella (26.97%), *Clostridium* (20.85%), and *Anaerosalibacter* (14.01%) on day 60 of decomposition. The relative abundance of *Bacillus* significantly increased on day 30 in all burial set-ups and decreased on day 60 of decomposition. On day 60, *Pseudogracilibacillus* was abundant in UA (27.81%) and SA (39.08%), while *Lentibacillus* was abundant in UAn (32.26%) and SAn (27.29%).

**Figure 8:**
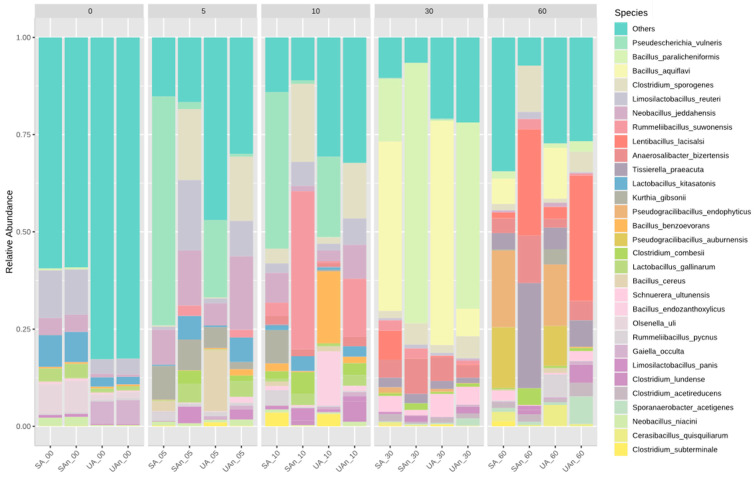
Relative abundance of the top 30 most abundant species during decomposition of poultry carcass. “Others” represent taxa with counts <10. UA, unsterilized soil–aerobic condition; SA, sterilized soil–aerobic condition; UAn, unsterilized soil–anaerobic condition and SAn, sterilized soil–anaerobic condition. The 0, 5, 10, 30, and 60 represent the sampling period.

At the ASV (species) level, the results showed that the bacterial community composition varied in different burial set-ups throughout the decomposition process (Figure 8). During the initial day, we identified that *Limosilactobacillus reuteri* was predominant in all burial set-ups. On day 60, the relative abundance of *Pseudogracilibacillus endophyticus* under aerobic conditions (UA and SA) was high compared to other species. On the other hand, *Lentibacillus lacisalci* dominated the community on day 60 in the burial set-up under anaerobic conditions (UAn and SAn). LEfSe analysis revealed that *Bacillus cereus* and *Gaiella occulta* were the species that showed significant increase in abundance in UA and UAn burial set-ups, respectively (*p* < 0.05) (Figure S2). In terms of day of decomposition, *Megamonas hypermegale*, *Blautia glucerasea*, and *Cuneatibacter caecimuris* were significantly higher on day 0 both in SA and SAn burial set-ups (*p* < 0.05). *Neobacillus jeddahesis* significantly increased at day 5 in UAn and SAn; *Bacillus kyonggiensis* at day 10 in SA burial set-up, while *Schnuerera ultunensis* and *Urmitella timonensis* at day 30 in both UA, SA, and UAn burial set-ups. In addition, *Tissierella praeacuta* and *Anaerosalibacter bizertensis* were significantly abundant on day 60 in SAn burial set-up; *Sporanaerobacter acetigenes* and *Clostridium acetireducens* in UAn; *Cerasibacillus quisquiliarum* in UA and *Lentibacillus lacisalci* were significantly high in abundance on day 60 of decomposition in UAn and SAn burial set-ups (*p* < 0.05).

## 4. Discussion

In this study, we investigated if the moisture content and pH were affected during the decomposition of poultry carcasses in vitro. The study also employed 16S rRNA Illumina MiSeq high-throughput sequencing using the ASV approach method to determine the changes in the bacterial communities in different burial set-ups of decomposing poultry carcasses.

The results of the study revealed that moisture content was higher in the burial set-ups under anaerobic conditions than those in aerobic conditions. This suggests that during the decomposition under anaerobic conditions, the water from the decomposing material was extracted, and moisture was evaporated and accumulated within the container. Several studies reported that the moisture content during decomposition increases as the temperature increases [35,36]. Since there was no oxygen supply in the anaerobic burial set-ups, the heat increased and accumulated inside the container, hence, the bound water in the decomposing body was released and moisture content increased [35]. At this period, the microbial activity would increase, as well as the production of enzymes by the microorganisms which aids in the decomposition of the carcasses.

In terms of pH, the pH varied in each burial set-up and across time. We observed a higher pH in burial set-ups under aerobic conditions compared to those in anaerobic conditions. Several studies reported that an increase in pH is due to the breakdown of nitrogen-containing organic matter, which leads to the accumulation of NH_3_ that dissolves in moisture to form alkaline NH_4_^+^ [37,38]. Thus, the increase in pH may be related to the moisture content and is important during carcass decomposition. In an actual livestock burial site, there is a lack of oxygen during the decomposition process and as a result the moisture content will increase and produce a large amount of leachate which may slow down the decomposition process. Meanwhile, a decrease in pH is attributed to the reduced production and increased release of NH_3_ and the subsequent release of ions [35,37].

The microbial diversity can be determined by several factors including physical, chemical, and biological characteristics of an ecosystem [39,40]. Carcass decomposition is directly associated with the alteration of bacterial community due to the breakdown of tissue, which releases an ammonia-rich, high-nutrient fluid that alters both the pH and nutrient content in the soil [41,42,43]. Microorganisms are key players during the decomposition of carcasses [11,44,45,46]. The Chao1 and Shannon index varied between different burial set-ups and across different sampling days during decomposition. Our findings demonstrated that the Chao1 and Shannon index decreased starting from day 5 of decomposition. This could be due to the different burial conditions and effect of the soil moisture and pH during decomposition [10,11,46,47]. In addition, we found that more bacterial taxa were found in the burial set-ups with unsterilized soil (UA and UAn) compared to burial set-ups with sterilized soil (SA and SAn). This indicates that the removal of intact microbes in soil by sterilization treatment significantly affected the diversity during the decomposition of poultry carcasses [11]. However, the burial set-up with unsterilized soil decomposed under anaerobic conditions (UAn) had a higher Chao1 and Shannon index compared to other burial set-ups at day 60. We speculate that as the decomposition progressed, the environment became anaerobic leading to an increase in anaerobic microorganisms. The breakdown of tissue is mainly dominated by anaerobic autolysis and later by microbe and insect infiltration [48], which could explain the dominance of anaerobic microorganisms. Furthermore, we observed that as pH increased during decomposition, the bacterial species present in the burial set-ups was decreased. The trend in pH was similar to the changes seen in bacterial communities, indicating that the bacterial communities were influenced by the pH. According to Rousk et al. [49], the relative abundance and diversity of bacteria were positively associated due to the narrow pH ranges for optimal growth of bacteria. Moreover, the survival of microbes is dependent on the pH, thus, when pH increased, the microbes which can survive at a higher pH become dominant [50]. Several studies reported that the pH of the soil is an important abiotic predictor associated with bacterial diversity [50,51,52,53]. Bacterial communities are more diverse in soils with near-neutral pH than in acidic or alkaline soils [50,51]. We observed that the alpha diversity was highest in all burial set-ups in the initial day than those found in other periods. In addition, these samples had near-neutral pH (6.5 ± 0.5) than the other samples. Likewise, the sterilization treatment of soil had a role in the diversity of microorganisms during the decomposing carcasses. Our findings were in accordance with the study of Lauber et al. [11], wherein a decrease in the alpha diversity and shift in the taxonomic composition was reported in the burial set-up with sterilized soil. These results suggested that the soil bacterial community was affected when the soil microbes were removed by sterilization treatment. The presence of these soil microbes can influence the decomposition process. These microbes can be characterized and utilized in order to promote the decomposition of livestock carcasses in an actual burial site. In addition, when comparing the burial set-ups by oxygen condition, the Chao1 in the unsterilized soil-anaerobic condition burial set-up (UAn) was higher compared to other set-ups. Microbial studies suggest that anaerobic bacteria flourish at most times during decomposition. According to Janaway [54], aerobic bacteria flourish during the early stages of the decomposition process as there is oxygen present within the body. Furthermore, as the microbial population increases, the accumulation of gases during the decomposition process makes the environment anaerobic which prompts the microbial community to shift [54].

Based on the PCoA, the comparison of bacterial communities between different burial set-ups at each time point showed significant variation. The bacterial species during early decomposition differs in the late decomposition. During the initial day, the bacterial community in the unsterilized soil burial setups (UA and UAn) and the sterilized soil burial setup (SA and SAn) were different from each other. This is probably due to the sterilization treatment which eradicated the intact microbial community in the soil. Similarly, a decomposition study by Lauber et al. [11] reported that bacterial communities of carcasses buried in soil with an intact microbial community and soil without an intact microbial community vary from each other. Moreover, several studies reported that bacterial communities vary significantly at different stages of decomposition [55,56]. However, as the decomposition progressed, we observed changes in the bacterial species present at each period across all burial set-ups. Based on these results, it showed that the presence of an intact microbial community in soil and oxygen availability during carcass decomposition influenced the bacterial community in the burial set-ups. Meanwhile, samples from days 5 to 60 showed a close association and were clustered by unsterilized or sterilized soil. This indicates that during this period, the burial set-ups that were clustered together had a similar bacterial composition. We speculate that these might be related to the moisture content and pH during the decomposition process, wherein it showed a stable pattern from days 30 to 60. The changes in the moisture content and pH during this period influenced the bacterial composition in the burial set-ups. Since the moisture content and pH were relatively stable at this point, we hypothesize that the bacterial community towards the end of decomposition also showed similar taxonomic composition.

Firmicutes, Actinobacteria, and Proteobacteria were the predominant phyla during carcass decomposition in all burial set-ups. Similar to other studies, these phyla were reported to be associated with the decomposition of carcasses [43,46,52,57,58]. Variation in the taxonomic composition was observed during the decomposition process. The changes in bacterial communities suggest that various bacterial species play a role during the decomposition period. During early decomposition, Proteobacteria and Actinobacteria were abundant; however, Firmicutes dominated the community as the decomposition process progressed. A decomposition study by Pechal et al. [46] showed that Proteobacteria declined in abundance, while Firmicutes increased in abundance. In this study, we observed that the abundance of Proteobacteria decreased on day 10, while the abundance of Firmicutes increased as the decomposition progressed. The taxa within Proteobacteria are commonly associated with the spoiling of meat and have been found on the hides of slaughtered animals [46]. Proteobacteria are common in soil and play an important role in the decomposition of fats and carbohydrates [57]. Meanwhile, Firmicutes are associated with the gut microbiome [58] and soil communities [52]. Firmicutes are involved in reducing large macromolecules, such as proteins, complex fats and polycarbohydrates to their building blocks [58]. Thus, they are more prominent during active decomposition [43,59]. Furthermore, during the initial day of decomposition, we observed that Firmicutes became more abundant in the burial set-ups with sterilized soil than in burial set-ups with unsterilized soil. Similar to other studies [43,57,58,59], Firmicutes gradually increase during the early process of decomposition (day 5), whereas Proteobacteria decrease as decomposition progressed. On the other hand, Actinobacteria are widely distributed in both terrestrial and aquatic ecosystems, especially in soil, where they play an important role in the recycling of refractory biomaterials through the decomposition of complex mixtures of polymers in dead plants, animals and fungal materials, such as chitin, keratin, and lignocelluloses [60,61].

The bacterial community at the genus level revealed that *Bacillus*, *Clostridium*, *Pseudescherichia*, *Lactobacillus*, *Rummeliibacillus*, *Limosilactobacillus*, *Neobacillus*, *Lentibacillus*, *Pseudogracilibacillus*, *Anaerosalibacter*, *Gaiella*, and *Sporanaerobacter* were among the common genera found in the burial set-ups. These microbes are potentially pathogenic in soil samples during carcass decomposition. In this study, the abundance of *Lactobacillus* was high during the early days and decreased as decomposition progressed. Similarly, Wang et al. [62] reported a sharp decrease in the abundance of *Lactobacillus* after 16 days of composting poultry carcasses. Members of *Lactobacillus* are associated with the decomposition of lipids and complex carbohydrates associated with animal carcasses [62]. Meanwhile, *Clostridium* was detected in the early period of decomposition in burial set-ups in anaerobic conditions. *Clostridium* species are ubiquitous anaerobic putrefactive bacteria that can be found in various environments and are potentially pathogenic [54,63,64]. Several decomposition studies have reported that the increase in abundance of *Clostridium* is due to the anaerobic layer that develops around a carcass during decomposition [23,65]. *Clostridium* species play an important role in biomass digestion as they synthesize a wide variety of extracellular enzymes which aids in the degradation of various compounds, such as carbohydrates, amino acids, alcohols, amino acids, or purines [23,65,66,67,68]. In the present study, *Gaiella occulta* was found abundant in the burial set-ups with unsterilized soil. *Gaiella* sp. which belongs to Actinobacteria were commonly found in soil [69,70]. This suggests that Gaiella occulta was intact in the unsterilized soil since this species was not found in the burial set-ups with sterilized soil. Meanwhile, *Megamonas hypermegale*, *Blautia glucerasea*, and *Cuneatibacter caecimuris* were found in SA and SAn burial set-ups during the initial day. These microorganisms are reported to be associated with the poultry intestinal microbiota [71,72,73,74]. *Pseudescherichia vulneris* is an environmental microorganism that can colonize humans and animals [75,76]. *P. vulneris* was reported as one of the contaminants in slaughtered poultry carcasses [77]. *Rummeliibacillus* sp. and *Neobacillus* sp. were also detected in the decomposing poultry carcasses in the different burial set-ups, however, very little is known about these microorganisms and their involvement in the carcass decomposition. *Rummeliibacillus suwonensis* is a Gram-positive, facultatively aerobic, rod-shaped, non-motile, terminal spore-forming bacterium which was first isolated in soil from Suwon, Korea [78]. *Neobacillus jeddahensis* is an aerobic, Gram-positive, rod-shaped, mesophilic bacterium that was isolated from the feces of a man from Jeddah, Saudi Arabia [79]. *Limosilactobacillus reuteri* was found in all burial set-ups, which indicates that this species originated from the animal. *L. reuteri* is a bacterium that is found in a variety of natural environments including different body sites, such as the gastrointestinal tract, urinary tract, and skin in a human and a large number of mammals [80]. *Sporanaerobacter acetigenes* is a strictly anaerobic, moderately thermophilic, and sporulating bacteria. *Sporonaerobacter* sp. are commonly observed in anaerobic utilization of municipal wastes, activated sludge, and decomposition of entombed pigs [81]. They are important members of the bacterial community for the destruction of the protein fraction of complex substrates in the form of volatile fatty acids (VFA). *Anaerosalibacter bizertens* is an anaerobic, spore-forming, thermophilic bacterium that was isolated from sludge and is reported to be a reducing species during the decomposition process [64,82]. We detected a significantly high abundance of this bacterium in the burial set-ups under anaerobic conditions (UAn and SAn) at day 60 of decomposition.

At the end of the decomposition period, the prevalent genus for burial set-ups under aerobic conditions is *Psedogracillibacillus*, while the most prevalent in the burial set-up under anaerobic conditions is *Lentibacillus*. *Pseudogracilibacillus* is an aerobic, spore-forming, gram-positive bacterium [83]. We detected *Pseudogracilibacillus endophyticus* in the burial set-ups under aerobic conditions (UA and SA), this suggests that this bacterium may be positively associated with aerobic conditions during decomposition. *Lentibacillus* is an aerobic or facultatively anaerobic, gram-variable, endospore-forming, moderately halophilic bacterium [81,84]. *Lentibacillus* was among the dominant bacteria detected in a composting system along with other anaerobic, thermophilic, endospore-forming, and/or halophilic gram-positive bacteria, such as *Pelotomaculum*, *Clostridium*, and *Caldicoprobacter* [45]. In this study, we detected a high abundance of *Lentibacillus* *lacisalsi* in the burial set-up under anaerobic conditions (UAn and SAn). This could indicate that this bacterium may be positively associated to an anaerobic condition during poultry carcass decomposition. *P. endophyticus* and *L. lacisalsi* both belong to Firmicutes; however, it is unclear at this time on their specific involvement in the carcass decomposition. These species of bacteria may be associated in the soil and have a role in decomposition with the metabolites they produce. The changes in the abundance at genus and species level could be attributed to the availability of oxygen rather than the soil condition as time goes by in each burial set-up.

## 5. Conclusions

Identification of microorganisms and changes in the community structure is important in order to determine the species involved and their possible role in the community during carcass decomposition. Our findings suggested that the presence of microbes in soil and oxygen availability significantly influenced the changes in moisture content, pH, bacterial abundance, and community composition during the process of decomposition of carcasses in vitro. This study provided plausible information on the possible bacterial species involved in the decomposition of poultry carcasses. Also, this study could be used to utilize potential microbes to increase the decomposition rate of animal carcasses and antagonistic action against contagious animal pathogens, such as avian influenza under actual burial conditions.

## Data Availability

The data presented in this study are available on request from the corresponding author.

## Supplementary Materials

The following are available online at https://www.mdpi.com/article/10.3390/ani11102937/s1, Table S1: Changes in the moisture content (%) and pH during decomposition of poultry carcasses in different burial set-ups, Figure S1: Rarefaction curves constructed based on the number of amplicon sequence variants (ASVs) found in each sample, Table S2: Alpha diversity indices during decomposition of poultry carcasses in different burial set-ups, Table S3: Taxonomic classification of shared bacterial ASVs present in SA, UA, SAn, and UAn samples, Table S4: Taxonomic classification of bacterial ASVs present only in SA, UA, SAn, and UAn samples, and Figure S2: Linear discriminant analysis Effect Size (LEfSe) of significant ASVs at species level that significantly changed across (a) each burial set-up and (b) time of decomposition.
