# Supplementary material for: Impact of Soil Microbes and Oxygen Availability on Bacterial Community Structure of Decomposing Poultry Carcasses
Source: Animals (Basel). 2021 Oct 11;11(10):2937. doi: 10.3390/ani11102937 (PMC8532636; doi:10.3390/ani11102937)
Supplement: Supplementary file 1 [file animals-11-02937-s001.zip › animals-1389998_Supplementary figures 09.14.pdf]

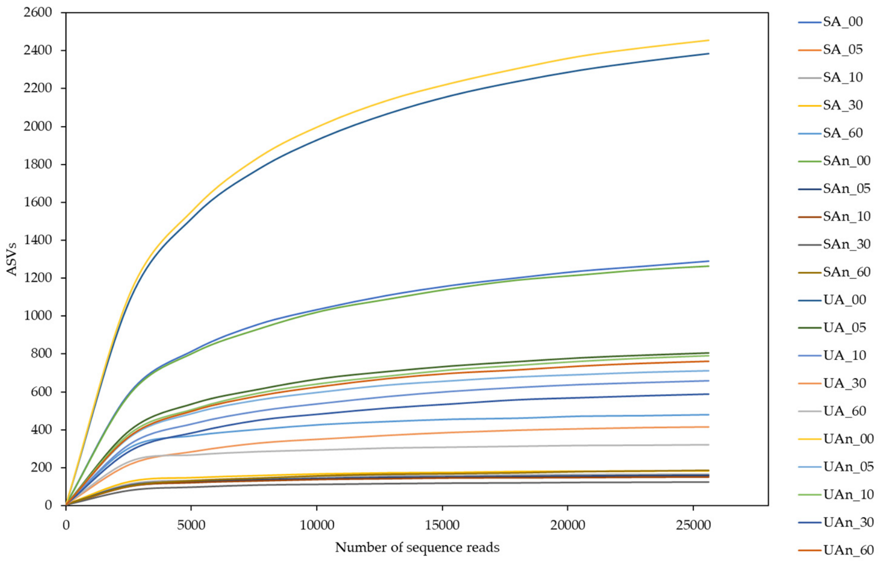

Figure S1. Rarefaction curves constructed based on the number of amplicon sequence variants (ASVs) found in each sample.

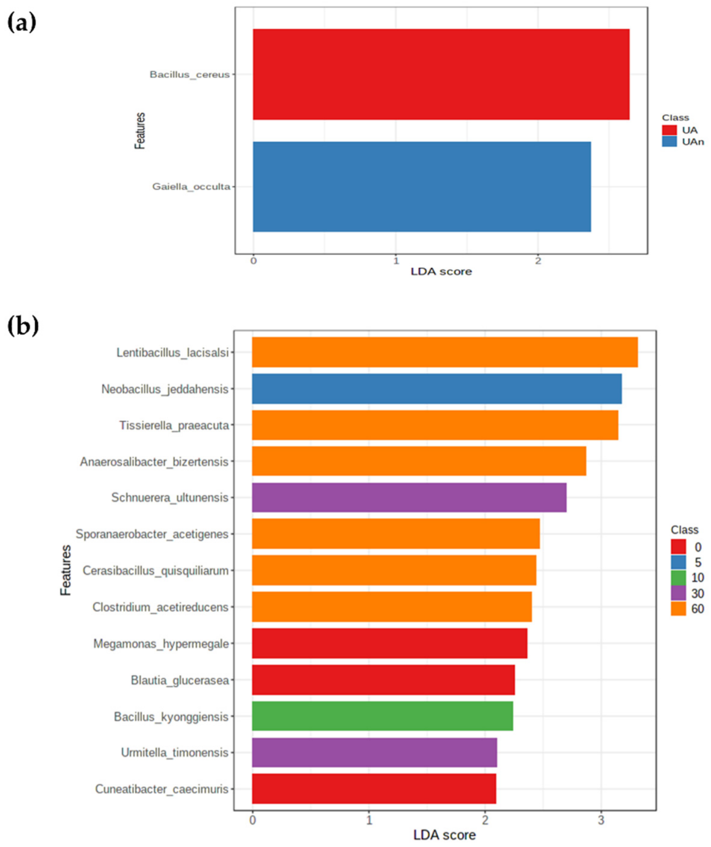

Figure S2. Linear discriminant analysis Effect Size (LEfSe) of significant ASVs at species level that significantly changed across (a) each burial set-up and (b) time of decomposition. LefSe was used to generate the LDA effect size with p value cut-off of 0.05 and LDA score of > 2.0.
